# Draining the Pleural Space: Lymphatic Vessels Facing the Most Challenging Task

**DOI:** 10.3390/biology11030419

**Published:** 2022-03-10

**Authors:** Eleonora Solari, Cristiana Marcozzi, Chiara Ottaviani, Daniela Negrini, Andrea Moriondo

**Affiliations:** Department of Medicine and Surgery, School of Medicine, University of Insubria, 21100 Varese, Italy; eleonora.solari@uninsubria.it (E.S.); cristiana.marcozzi@uninsubria.it (C.M.); cottaviani1@uninsubria.it (C.O.); daniela.negrini@uninsubria.it (D.N.)

**Keywords:** pleural cavity, lymphatic vessel, diaphragm, chest wall, lung, breathing

## Abstract

**Simple Summary:**

Fluid drainage operated by lymphatic vessels is crucial for a proper volume homeostasis of body compartments. This role is particularly relevant for the pleural cavity, where the hydraulic pressure of the pleural liquid is very subatmospheric and fluid filtering from the blood capillaries into the pleural space must be continuously removed to keep the pleural space volume low and to prevent accumulation of liquid causing impairments of the respiratory mechanics. In order to accomplish this task, lymphatic vessels of the pleural side of the diaphragm and those lying on the pleural surface of the chest wall must possess a negative intraluminal pressure which has to vary during the respiratory cycle to follow the similar variations occurring to the pressure of pleural liquid. This review focuses on the in vivo pressure measurements performed in sedated animal models to understand how these lymphatic networks can accomplish this complex but pivotal role.

**Abstract:**

Lymphatic vessels exploit the mechanical stresses of their surroundings together with intrinsic rhythmic contractions to drain lymph from interstitial spaces and serosal cavities to eventually empty into the blood venous stream. This task is more difficult when the liquid to be drained has a very subatmospheric pressure, as it occurs in the pleural cavity. This peculiar space must maintain a very low fluid volume at negative hydraulic pressure in order to guarantee a proper mechanical coupling between the chest wall and lungs. To better understand the potential for liquid drainage, the key parameter to be considered is the difference in hydraulic pressure between the pleural space and the lymphatic lumen. In this review we collected old and new findings from in vivo direct measurements of hydraulic pressures in anaesthetized animals with the aim to better frame the complex physiology of diaphragmatic and intercostal lymphatics which drain liquid from the pleural cavity.

## 1. Lymph Draining and Propulsion

The lymphatic system drains liquid, macromolecules and cells from the surrounding interstitial space and serosal cavities, to guarantee the proper tissue fluid balance [1]. Lymph formation happens first into lymphatic capillaries, named “initial lymphatics”, which are blind-ended vessels originating in peripheral tissue, devoid of lymphatic muscle cells (LMCs). Those vessels are lined by a single layer of overlapping endothelial cells (LECs) forming primary unidirectional valves, allowing lymph entry and preventing its backflow into surrounding tissue [2,3,4,5]. The forces required for lymph formation and propulsion are modelled according to the Starling’s Law Equation (1) [6]. Hydraulic and colloidosmotic pressure gradients are the main determinants of lymph flow (*J_lymph_*) across the capillaries’ wall and through the lymphatic network

(1) $$J_{lymph}=-L_{p}\cdot S\cdot \left(\Delta P_{TM}-\sigma \cdot \Delta P_{\pi }\right)$$

where *L_p_* is the endothelial permeability to water coefficient, *S* the surface area of fluid exchange, *σ* is the capillary reflection coefficient to solutes (ranging from 0 to 1), and Δ*P_TM_* and Δ*P_π_* the hydraulic and colloidosmotic pressure gradients between the lymphatic capillary lumen and the interstitium (Δ*P_TM_* = *P_L_* − *P_in_*; Δ*P_π_* = *P_π_*_,*L*_ − *P_π_*_,*in*_).

In lymphatic capillaries *σ* is ~0 and interstitial proteins are not restricted to the interstitium. Therefore, colloidosmotic pressure is almost the same in both interstitial space and lymphatic lumen (*P_π_*_,*L*_ = *P_π_*_,*in*_) thus the colloidosmotic contribution to lymph formation is null resulting in the modified Starling’s Law Equation (2)

(2) $$J_{lymph}=-L_{p}\cdot S\cdot \left(\Delta P_{TM}\right)$$

Consequently, the main driving force for lymph draining only relies on the net Δ*P_TM_* pressure gradient. Particularly, *J_lymph_* is directly dependent upon the plasma filtered from blood capillaries into the interstitium (interstitial fluid volume) and the mechanical compliance of the receiving space: the more fluid enters the interstitium, the higher *P_in_* (particularly in stiffer tissues), the greater fluid volume is drained by lymphatics, whose *P_L_* is typically subatmospheric.

Due to anchoring filaments [7], lymphatic capillaries are tightly attached to the extracellular matrix [6,8] and tissue displacement causes extrinsic forces to cyclically expand and compress lymphatic vessels. This, in turn, affects Δ*P_TM_*, driving lymph formation. Lymph is then propelled through larger vessels, named “collecting lymphatics”, endowed with a more structured vessel wall, also surrounded by LMCs displaying unique features [9], in which intraluminal competent valves can be identified separating adjacent lymphangions (i.e., the functional pump units of the lymphatic system) and preventing massive lymph backflow [4,8,10]. Lymph progression is then due to a net intraluminal hydraulic pressure gradient acting across the trans-valve region (Δ*P_tv_*). However, the movement of lymph across the intraluminal valves has proven to be more complex than what can be expected from a direct analogy with cardiac valves. Early in vivo micropuncture studies measured a Δ*P_tv_* of ~1–1.5 cmH_2_O [11] across the valve to force it to open and let lymph flow through it in the direction imposed by the pressure gradient. More recently, *P_L_* measurements were performed while challenging mesenteric lymphatics with different pressure regimens applied to the vessel as a whole and across the intraluminal valves. Data showed that multiple gating patterns can be present, depending on the overall mean baseline *P_L_* inside the vessel and on the distension of the vessel wall [12]. As an example, valve closure may occur with an adverse Δ*P_tv_* as low as 0.1 cmH_2_O for baseline intraluminal pressures below 2 cmH_2_O, but a Δ*P_tv_* higher than 2 cmH_2_O is required to force the intraluminal valve to close with a baseline intraluminal pressure of 10 cmH_2_O or above. Moreover, pressure ranges for valve opening and closure are not symmetrical, and this causes valves to be biased in an open position that can have multiple physiological implications and can also lead to a limited lymph backflow across an open valve. The presence of an actual lymph backflow across an intraluminal valve biased in an open position has been independently confirmed in separate works by Dixon et al. and our group [13,14] in video recordings of the motion of lymphocytes or particles suspended in the lymph. By a step-by-step progression along adjacent lymphangions and through lymph nodes, lymph is then propelled centripetally. The rise in prenodal pressure due to intrinsic and extrinsic forces is dissipated into the lymph nodes, as hypothetically depicted by Aukland and Reed (see [6], Figure 13). Nevertheless, lymph is propelled against an overall hydraulic pressure gradient to the blood venous system [11,15]. Lymphatic spontaneous phasic contractions (intrinsic forces) [16] arising in collecting vessels walls combined to extrinsic mechanisms drive lymph propulsion centripetally, critically affecting intraluminal pressure gradients. Pacemaker activity governing the spontaneous contractions of the lymphatic muscle [9,17,18,19,20] typically spreads from a subset of LMCs in the vessel wall and propagates along the lymphatic network through gap junctions between LMCs and/or LECs [15]. Lymph is then allowed to unidirectionally flow towards the lymph node chain and merge into the thoracic duct and right lymph duct, eventually emptying into the blood circulatory system through the subclavian veins.

## 2. The Pleural Space

The pleural space in the thoracic cavity is a very peculiar serosal compartment whose integrity is vital to allow for a correct, physiological breathing. The mechanical properties of lungs and chest wall are set so that each of the two structures tends to reach its mechanical resting volume. These volumes are located at two very different values, as lungs tend to the very low minimal air volume (~500 mL, below the in situ residual volume corresponding to zero vital capacity) whereas the chest wall tends to a value ~80% of the vital capacity [21]. In so doing, they both tend to increase the pleural cavity volume, which, filled with pleural liquid, reaches subatmospheric pressure values (*P_liq_*, pleural hydraulic pressure, analogous to *P_in_* for the pleural cavity) during normal, spontaneous breathing. On average, in humans, at end-expiration at the height of the right atrium the pleural liquid has a pressure of ~–5 cmH_2_O, which becomes more negative during inspiration, reaching the lowest value ~–30 cmH_2_O at end-inspiration at total lung capacity. Pleural *P_liq_* only becomes positive during forced expiration, reaching values in excess of ~200 cmH_2_O during maximal expiratory effort, and lower than ~–150 cmH_2_O during maximal inspiratory effort, in absence of air flow. The negative *P_liq_* ensures that a proper mechanical coupling exists between the chest wall and the lungs, but it meanwhile implies that the removal of liquid filtered from capillaries and surrounding tissues is more difficult to achieve—Equation (2).

In the following sections we will review old and newer literature to frame the peculiar role those lymphatic vessels of the thoracic cavity wall must exert in order to attain a suitable draining capability, thus assuring the homeostasis of pleural liquid volume and pressure for a physiological, normal spontaneous breathing. Relevant physiological data are based on lymphatic intraluminal and tissue hydraulic pressure measurements, performed both in the pleural cavity and/or lymphatic vessels of the diaphragm and the thoracic wall. Most of these works have been performed in not so recent years but still extremely important to those interested in understanding how complex and delicate is liquid and volume homeostasis in the pleural cavity.

## 3. Lymphatic Vessels of the Diaphragm

Lymphatic vessels of the diaphragm remove liquid filtered from blood capillaries into the interstitial space, as occurs in all skeletal muscle tissue, but also allow fluid homeostasis of the pleural (and peritoneal) cavities, due to their subatmospheric intraluminal pressure. On the pleural side, lymphatic vessels are organized in linear vessels, with a parallel or transverse arrangement with respect to the skeletal muscle fibers orientation, or in complex merging structures (lymphatic loops) [22,23]. The excess fluid removal must be tightly controlled, to minimize pleural fluid volume thus assuring a proper physiological lungs–chest wall mechanical coupling. Therefore, diaphragmatic lymphatics on the pleural side play a critical role in balancing pleural fluid dynamics, since amiloride-mediated block of their intrinsic spontaneous contractility reduces the total pleural fluid egress by ~40% [24].

Unlike pleural lymphatics, on the peritoneal side, lymphatic vessels are radially arranged from the abdominal wall to the central tendon [25]. They primarily remove peritoneal fluid, but also pathogens and/or immune cells related to inflammatory state affecting the viscera, such as the gastrointestinal tract or the urogenitary system.

**Figure 1:**
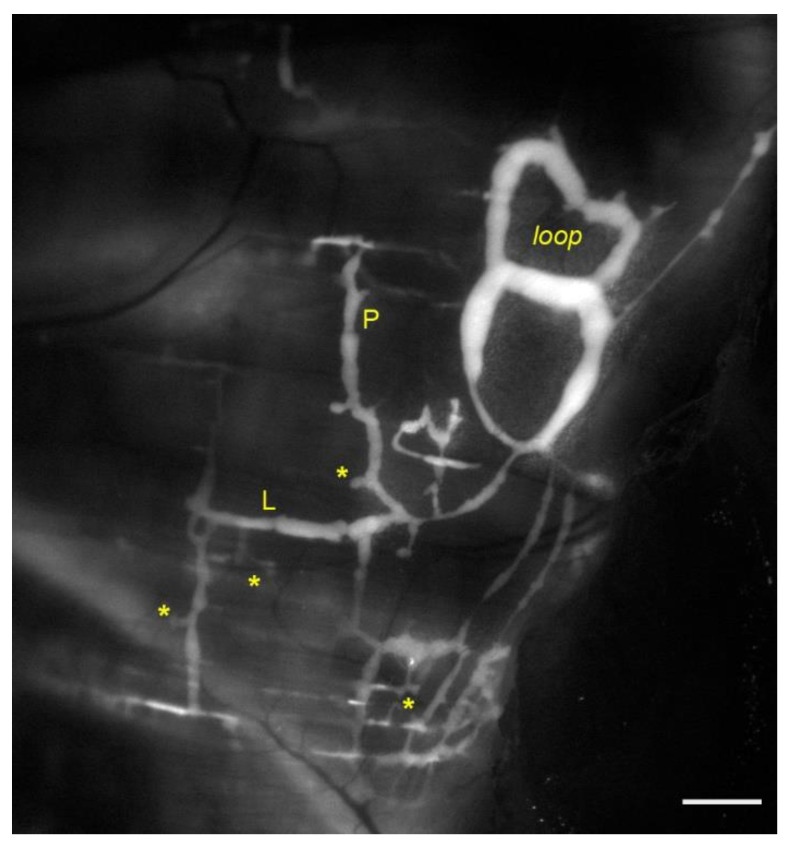
Representative image of lymphatic vessels organization on the pleural side of rat diaphragm, after the in vivo staining with a FITC-conjugated fluorescent tracer. Lymph enters lymphatic lacunae (**asterisks**) and then is propelled through vessels longitudinally (**L**) and/or perpendicularly (**P**) arranged with respect to the skeletal muscle fibers orientation. Lymphatic collectors located at the muscle periphery, next to the costal margin, are typically organized in complex loop structures (loop) and display intrinsic contractility. Scalebar 1 mm.

Lymph drained from serosal cavities enters the diaphragmatic network through mesothelial stomata, intercellular enlargements forming elliptical or round openings between mesothelial cells, having diameters in the range 0.5–20 µm [26]. Then lymph reaches flat submesothelial lymphatic lacunae (Figure 1, asterisks), which can be considered as larger lymphatic capillaries. Lacunae are located beneath the mesothelium and above the skeletal muscle layer and are unevenly distributed. In rabbits, they are densely located in both the tendinous regions, particularly on the peritoneal side of the diaphragm. Conversely, lymphatic lacunae are scanty in the muscular regions, of both sides [27]. Consistent to lacunae distribution, lymphatic stomata are also mainly distributed in the pleural and peritoneal tendinous regions of the diaphragm [26]. On the contrary, in mice and rats, stomata are restricted on the muscular region. Submesothelial lacunae then empty into transverse lymphatic vessels draining into collecting lymphatics deeper in the interstitium. Fluid and solutes removal from the interstitial space between diaphragmatic skeletal muscle fibers occurs in lymphatic capillaries, also emptying into larger collectors [28]. Lymph is then routed towards the mediastinal lymph nodes, merging to the blood stream via the thoracic duct. Intrinsic and/or extrinsic forces deeply affect lymphatic vessels’ draining and propulsive capability. Regarding extrinsic forces, the contraction of diaphragmatic skeletal muscle fibers during the respiratory cycle plays a pivotal role in determining lymphatic function. In humans the average resting respiratory rate (RR) is about 12–16 min^−1^, ref. [29] whereas in rodents it is significantly higher (about 85 min^−1^, in rats, reaching values up to 230 min^−1^, in mice) [30,31]. Given that at each respiratory act the diaphragm cyclically contracts and relaxes, respiratory movements give rise to cyclical mechanical stresses to be transmitted to lymphatic vessels, which primarily affect Δ*P_TM_* and the intraluminal pressure gradient (Δ*P_Lymph_* = *P_L_*_,1_ − *P_L_*_,2_) between two lymphatic segments. The external stress transmission efficiency depends upon both the mechanical properties of the lymphatics’ wall and the surrounding tissue, being more effective in stiffer rather than more distensible regions [32]. Moreover, depending on vessels’ orientation with respect to the skeletal muscle fibers, extrinsic forces can either shrink or enlarge the vessel lumen, affecting lymphangions’ volume. Experiments performed on the rat spinotrapezius muscle showed that the contraction of skeletal muscle fibers by ~20% induced vessels compression of about 30%, whereas an opposite ~20% skeletal fibers stretch exerted a tensile stress expanding lymphatics lumen of about 40% [33]. Therefore, tissue compression and expansion deeply affect intraluminal and interstitial hydraulic pressure profiles. 

In vivo *P_L_* recordings performed on rat pleural vessels while inducing active diaphragmatic skeletal muscle contractions [34] revealed that in superficial, perpendicularly oriented (Figure 1, P) lymphatic vessels each contraction of the diaphragm reduces the diameter by ~39%. This in turn causes a decrease by ~12 cmH_2_O in *P_L_* in submesothelial vessels, maybe enhancing the pressure gradient (Δ*P_TM_*) driving force thus favoring lymph formation. Conversely it induces *P_L_* increase by ~11 cmH_2_O in deeper perpendicularly oriented collectors, favoring lymph propulsion. In longitudinally oriented vessels (Figure 1, L) diaphragmatic muscle contractions reduce the vessels’ length by ~30% whereas the lymphatic diameter enlarges by ~23%. In these longitudinally oriented lymphatics, *P_L_* decreases in both superficial submesothelial and deeper vessels by ~22.5 cmH_2_O and ~10.5 cmH_2_O respectively, thereby supporting further lymph propulsion. The different regional effect may also be explained considering that deeper lymphatics are entirely surrounded by stiffer skeletal or tendinous fibers, thus displaying a compliance ~2 order of magnitudes lower than superficial vessels. Hence mechanical stresses are better transmitted to those lymphatics, with only minor wall deformation [32]. 

Another source of extrinsic forces is the rhythmic tissue displacement due to heart activity, as cardiogenic movements induced by arterial pressure pulses propagate through neighbor regions, affecting the diaphragmatic lymphatic bed and the adjacent interstitium. Experiments performed on rats and rabbits [35] demonstrated a perfect in-phase cardiogenic effect in 33% of oscillatory pressure profiles of diaphragmatic *P_L_* and *P_in_*, as both displayed highest values overlapping systolic arterial pressure peaks, whereas the lowest values corresponded to the arterial diastolic pressure. However, in most cases *P_L_* and *P_in_* oscillations were out-of-phase with respect to each other and to the arterial pressure wave, displaying variable phase shift, further suggesting the existence of a very complex and anisotropic tissue stress distribution in the diaphragmatic dome. As a result, the cardiogenic activity can simultaneously sustain lymph formation and propulsion along the lymphatic network of the diaphragm. In fact, the overall data showed that in ~60% of cases Δ*P_TM_* sustained lymph formation during either systolic or diastolic phases, whereas in the other 40% of cases Δ*P_TM_* become transiently positive. In the latter case Δ*P_TM_* might possibly drive fluid backflow into the interstitial space but might sustain oscillatory lymph propulsion along lymphatic collectors.

**Figure 2:**
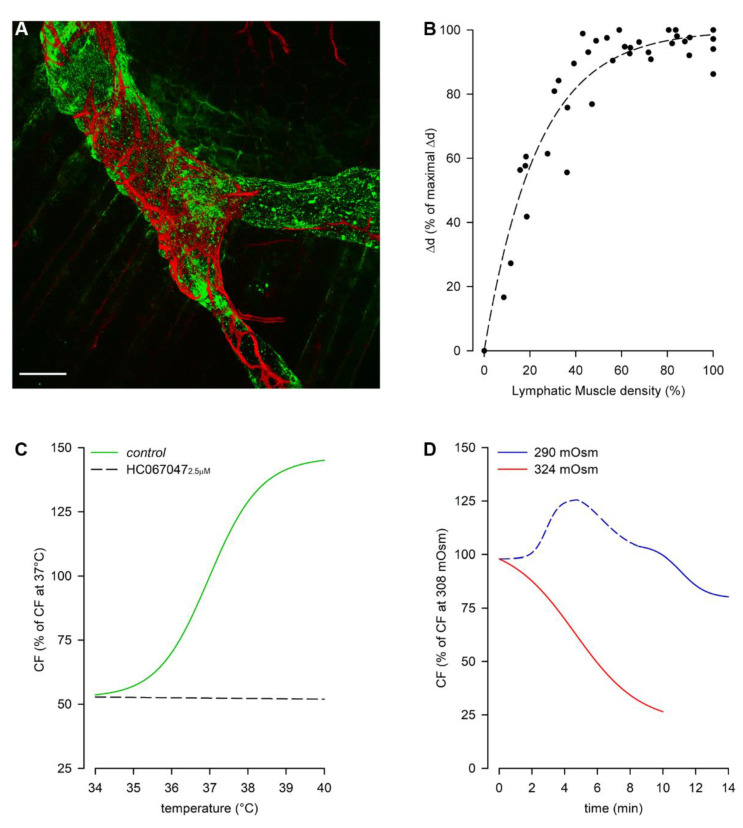
(**A**) Representative confocal image of a rat pleural diaphragmatic lymphatic vessel in vivo stained with FITC-dextrans (green signal) and whole mount stained for LMCs (red signal), highlighting the organization of the lymphatic muscle mesh surrounding the vessel (scalebar 100 µm). (**B**) Plot of rat diaphragmatic lymphatics intrinsic contraction amplitude (Δd) correlating to the density of lymphatic muscle in the vessels’ wall. (**C**) Plot of the dependence of rat diaphragmatic lymphatics intrinsic CF from temperature in the range 34–40 °C (green trace). Temperature-dependency is completely abolished by the selective TRPV4 channels antagonist HC067047 (2.5 µM, black dashed line). (**D**) Plot of osmolarity-induced modulation of rat diaphragmatic lymphatics intrinsic CF. The hyperosmolar environment (324 mOsm, red trace) induces a sigmoidal decease in CF. Hyposmolarity induces a two-phase response, as lymphatic vessels display an acute early CF increase (290 mOsm, blue dashed line) followed by a later CF decrease (blue solid line).

Diaphragmatic collecting lymphatics located at the far peripheral muscular region adjacent to the costal margin are typically organized in complex loops (Figure 1, loop) and are also endowed with a properly organized dense mesh of lymphatic muscle (Figure 2, panel A), which spontaneously contracts [23,36] aiding lymph propulsion. Those intrinsic spontaneous contractions rely on a “Funny”-like current (I_f_) due to HCN (Hypolarization-activated Cyclic Nucleotide-Gated) channels [37,38], as it occurs in the heart pacemaker, even if in other body districts other mechanisms have been described, related to Spontaneous Transient Depolarizations (STDs) [38,39]. In mammals, the HCN channel family consists of four members (HCN_1-4_), which are activated upon hyperpolarization, starting at a membrane potential of ~−50/−60 mV, and are permeable to both Na^+^ and K^+^ cations (being the ratio permeability P_Na_:P_K_ 1:3 to 1:5) [40]. Experiments performed on rat diaphragm [37] pointed out that collecting lymphatics display all HCN_1-4_ isoforms in their wall, and intrinsic pumping behaves similarly to the sinoatrial pacemaker [41] as it can be reduced by Cs^+^ application [38,42] or it can even be abolished by application of two well-known HCN channel blockers, Ivabradine [43] and/or ZD7288 [42]. 

In analogy to the cardiac cycle, the intrinsic contractile mechanism can be divided into an active systolic contraction followed by a diastolic relaxation, and it can be quantified in terms of contraction frequency (CF, cycles/min) and contraction amplitude (Δd, the difference between the end-diastolic and the end-systolic diameter lengths) [44]. Particularly, in rat diaphragmatic lymphatics, LDLs (low-density lipoproteins) application increases lymphatic CF even if, at variance to the cardiac pacemaker, such mechanism seems to be due to a shortening of the systolic rather than the diastolic phase [45]. 

Data from rabbits show that during the systolic phase Δ*P_Lymph_* increases up to ~10 cmH_2_O [46] in superficial pleural lymphatics, independently from vessel’s diameter, allowing a pressure gradient favoring lymph propulsion and preventing fluid stasis. However, during spontaneous LMCs contractions intraluminal valves are biased in an open position, and a modest lymph backflow often occurs [4,12], allowing an oscillatory lymph flow inside the vessel, which results to be very limited, at least in pleural diaphragmatic lymphatics [14]. Compared to intrinsic forces, extrinsic ones are more efficient and guarantee a proper one-way propulsion by forcing intraluminal valves into an open/closed cycle [34], preventing backflow. In fact, the extrinsically induced lymph progression along the vessel network is ~30 times longer than the one caused by a single intrinsic contraction. In the former case the mean lymph velocity is also significantly higher (~340 µm/sec vs ~18 µm/sec) even if lymph flow remains laminar, as confirmed by the very low Reynold’s number also for extrinsic pumping mechanism. Moreover, diaphragmatic muscle contractions also enhance lymph flow, as it reaches values 10-fold higher than the ones induced by lymphatic muscle spontaneous contractions [14].

Then, why is the intrinsic mechanism also needed in diaphragmatic lymphatics? It seems to be restricted to the muscular peripheral vessels [23], located in an area where the extrinsic forces due to respiratory movements are less effective, probably due to the anisotropic stress distribution in the diaphragmatic dome [47]. In fact, it is worth noting that lymphatic vessels located in the medial muscular region also possess LMCs, even if they do not spontaneously contract. In those vessels the lymphatic muscle seems to be longitudinally rather than circularly oriented with respect to the vessel’s path [23], not properly arranged for squeezing the vessel. Moreover, the abundance of LMCs in the vessel wall, beyond a certain level, may not be determinant for the contraction amplitude. The analysis of active vessel’s edges displacement as an index of Δd revealed that during the intrinsic systolic phase the contraction amplitude reaches a finite saturation limit for increasing LMCs density, not corresponding to complete occlusion of the lymphatic lumen (Figure 2, panel B) [45].

Spontaneous LMCs contractions seem to allow lymph recirculation into peripheral lymphatic loops, thus preventing fluid accumulation which may result in the development of oedema. Intrinsic contractility of those vessels can be modulated by local physical factors, such as tissue temperature and fluid osmolarity, as lymphatics rapidly adapt to changes in the surrounding environment (Figure 2, panel C, green trace) [48]. Rat diaphragmatic lymphatic vessels lay in the thermal core at 37 °C, and sense changes in temperature via the TRPV4 (Transient Receptor Potential channel Vanilloid 4) thermal sensors [49], as the specific antagonist HC067047 [50] blocks those receptors giving rise to a temperature-insensitive behavior (Figure 2, panel C, black dashed line). Conversely, specifically activating TRPV4 receptors through GSK1016790A [51] application results in a lymphatic contractile behavior mirroring the response to increasing temperature. Those vessels can quickly adjust CF to changes in tissue temperature, in the physiological range 35–39 °C, as it can occur during circadian oscillations of temperature, but also in case of fever or during physical exercise. They typically contract at a rate about 19 cycles/min, however at 35 °C CF reduces about −54% whereas it increases about +34% at 39 °C. Considering the whole temperature range, raising temperatures exert an increase in CF (positive chronotropic effect), while simultaneously reduce Δd (negative inotropic effect) and the stroke volume (SV, the lymph volume ejected at each single lymphatic contraction). However, as the chronotropic effect prevails, raising the temperature rapidly increases lymph flow [52].

A more complex response occurs when liquid osmolarity deviates from the isosmotic mean value of about 308 mOsm (rat normal plasma osmolarity range is 288–336 mOsm) [53]. When diaphragmatic lymphatics are exposed to a hyperosmotic environment (Figure 2, panel D) CF decreases in an osmotic-dependent manner (~−54% at 315 mOsm and ~−70% at 324 mOsm), which seems to represent a tissue fluid-saving mechanism. On the other hand, the exposure to hyposmotic interstitial fluid exerts a biphasic behavior, displaying an early transient CF increase (+~17% at 299 mOsm and +~34% at 290 mOsm, Figure 2, panel D, blue dashed line), which probably responds to the need of removing a greater volume of fluid filtered. Later, CF decreases attaining an osmolarity-independent steady plateau (range ~−18–23%, Figure 2, panel D, blue solid line). As in both hyper- and hypo-osmotic environments no inotropic effects can be found, changes in lymph flow are qualitatively similar to the ones in CF [53]. 

The coexistence of both intrinsic and extrinsic mechanisms driving lymph dynamics makes the diaphragm a very intriguing model to study the mechanisms of lymphatic functionality. A similar setting can be found in lymphatic vessels of the gastrointestinal tract, even if it seems that in those vessels intrinsic and extrinsic mechanisms could be more spatially segregated [54,55,56]. Conversely, in most tissues lymph formation and propulsion are typically related to one specific mechanism, as it occurs in highly moving tissues such as other skeletal muscles, lungs and the heart, in which lymphatic function mainly relies on extrinsic forces [8,33,57,58]. In lymphatic vessels immersed in soft tissues, almost completely devoid of tissue displacement, the lymphatic draining capability totally depends upon the intrinsic contractility of LMCs, which can be modulated by different mechanisms, also involving physical factors, inflammatory mediators and the autonomous system [59,60,61,62,63,64,65,66].

## 4. Pleural Intercostal Lymphatics

**Figure 3:**
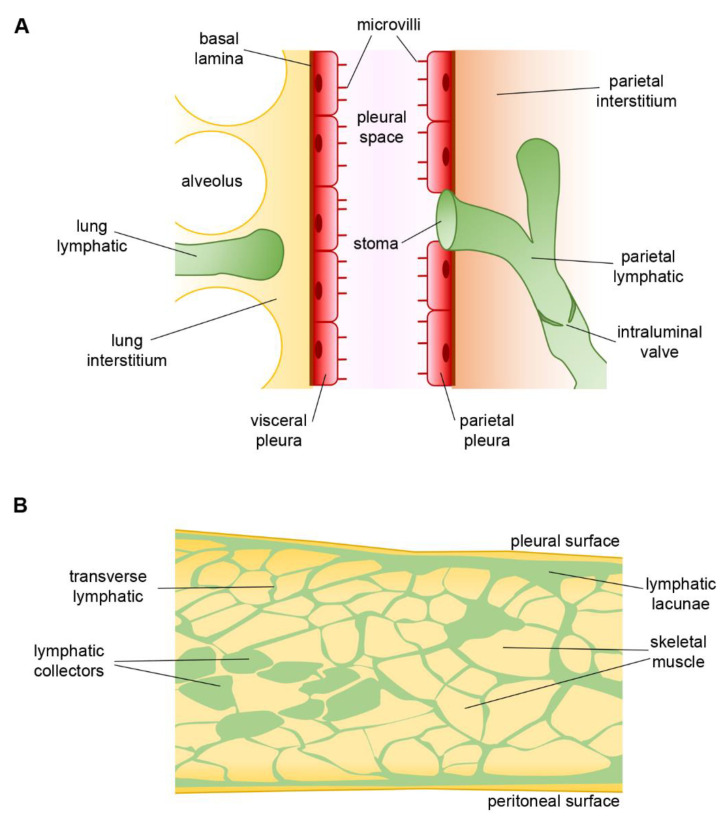
(**A**) Anatomical arrangement of parietal and visceral pleurae and the pleural space among them. Lymphatic stomata open directly into the pleural cavity. (**B**) Drawing of rat diaphragm cross section showing the reciprocal anatomical relationship among pleural and peritoneal submesothelial lymphatic lacunae, the transverse lymphatic network, and central lymphatic collectors (green). Skeletal muscle fiber bundles are shown in yellow and provide a strong extrinsic mechanical support to lymph propulsion during spontaneous breathing.

The pleural cavity is covered by parietal and visceral mesothelial pleura, which respectively cover the internal intercostal respiratory muscles and the lungs. Parietal pleural lymphatics of the intercostal spaces open directly in the pleural cavity, thus forming a direct funnel to the draining lymphatic system [67] (Figure 3, panel A). Those vessels might share some of the features of the diaphragmatic vessels, especially those related with the extrinsic forces sustaining lymph formation and propulsion. They are conveniently located in order to study how different ventilatory regimens may impact the lymphatic function. Indeed, these vessels are accessible for intraluminal pressure recordings once the intercostal muscle layers are removed, leaving exposed an intact pleura (pleural window) and thus a still-closed thoracic cavity. Larger lymphatic vessels can be visualized lying parallel and in proximity with the ribs and/or sternum and offer a suitable site for hydraulic pressures measurement [46].

During spontaneous breathing the simultaneous recording of *P_L_* and *P_in_* (taken from the tissue surrounding the lymphatic vessel under investigation) showed a variability of both, related to the phase of the respiratory cycle [68]. Overall, *P_L_* and *P_in_* reach their lowest values at the end of the inspiratory phase (about −28.5 and −16.5 cmH_2_O respectively), with *P_L_* remaining subatmospheric and below *P_in_* most of the times (end-expiratory mean values are about −3.4 cmH_2_O for *P_L_* and +4.2 cmH_2_O for *P_in_*). This in turn results in a Δ*P_TM_* favoring lymph formation in most vessels during the whole respiratory cycle, with an apparent prevailing role of tissue displacement over RR to drive the mean value of Δ*P_TM_* and thus lymph draining. However, in a small fraction of the intercostal vessels Δ*P_TM_* favors lymph propulsion for the entire respiratory cycle, whereas for some vessels their behavior changes from formation to propulsion in the transition between inspiration and expiration. Therefore, the spontaneous respiratory activity maximizes lymph formation while preserving lymph progression along the lymphatic network, both being mechanisms essential for a proper control of the pleural liquid volume.

The situation completely changes when the same pressure measurements are performed during controlled mechanical ventilation, even by setting respiratory parameters equal to spontaneous breathing. The major change happens in the *P_in_* and *P_L_* traces, since their values increase with increasing lung volume, reaching their maximal value at end-inspiration (about 38 cmH_2_O for both), and most notably *P_L_* becomes positive. The difference between intraluminal and tissue hydraulic pressures diminishes throughout the whole respiratory cycle and this in turn almost nullifies Δ*P_TM_*, with a severe impact on both lymph formation and propulsion. Moreover, an accurate analysis in the frequency domain of the various components of *P_in_*, *P_L_* and Δ*P_TM_* revealed that, while in spontaneous breathing all of them were dominated by the RR, during mechanical ventilation the relative power of the respiratory-driven frequency component of Δ*P_TM_* was greatly reduced and became almost as low as that of the cardiogenic oscillations in pressure [68].

Overall, in the transition from spontaneous breathing to mechanical ventilation, both draining and propulsive functions of costal lymphatics are severely depressed, leaving only the lesser functional cardiogenic activity to sustain a very limited lymph fluid removal from the pleural space. Thus, being *P_liq_* subatmospheric, the mechanical ventilation could increase the hydration state both of pleural space and lung tissues, causing the latent lung sub-oedematous condition often evident in patients mechanically ventilated at positive alveolar air pressures.

## 5. Lymphatic Vessels of Airways and Lungs

The anatomical details of the lymphatic system draining the lungs are well reported in literature [69,70]. Overall, it can be divided in lymphatics serving the upper airways, the tracheobronchial tree, and the lung parenchyma. Pulmonary lymphatics are organized into pleural vessels, located in close contact with the lung parenchyma, and interlobular or intralobular lymphatics of the loose connective tissue. The latter include bronchovascular, perivascular, peribronchiolar and interalveolar lymphatic vessels [71,72]. According to the lymphatic network craniocaudal distribution, lymphatic clearance routes change with lung height, as in the upper area of the lungs lymph formation prevails in bronchovascular lymphatics, whereas in the lower lung region it is predominant in subpleural/septal zones [73]. Overall, it is reported that lymph clearance is greater in lymphatics located in lower than in upper regions of the lung [74]. Pulmonary lymph flow progresses in two opposite directions: centrally towards the hilar lymph nodes and from the lung perimeter to the pleural region, considered a more efficient propelling route.

In humans, the lymphatic system begins to develop between the 6th and 7th week of pregnancy, whereas in mice it appears around the embryonic day 10 (E10.0). The well-known homeobox transcription factor Prox1 (Prospero homeobox 1) [75] and the growth factor VEGFC (Vascular endothelial growth factor C) [76] are typical LECs markers, playing a critical role in lymphatic vessels prenatal sprouting from embryonic veins and maturation [77,78]. Prox1 upregulates the expression of lymphatic-specific genes in LECs acting as a master regulatory gene leading to lymphatic commitment and maintenance [79,80]. VEGFC is a potent lymphangiogenic factor, thus knock out (KO) mice die at E15.5 due to fluid accumulation in tissues, whereas heterozygous display cutaneous lymphatic hypoplasia and lymphedema [81]. During embryogenesis VEGFC and VEGFR3 (Vascular endothelial growth factor receptor 3) [82] expression is involved in pulmonary lymphatics formation, critical for lung development. Indeed, mice lacking lymphatic vessels die at birth due to respiratory failure, despite a normally developed lung parenchyma and a proper amount of surfactant. However, the wet/dry ratio indicates the development of pulmonary oedema, significantly reducing the compliance of lung parenchyma and thickening the interstitium. As a result, mice are unable to inflate lungs at birth [83]. On the contrary, mice lacking the α9 integrin appear normal at birth, but they develop late-onset respiratory distress and die between 6–12 days after birth due to pleural fluid effusion and accumulation of liquid enriched in triglycerides and cholesterol (chylothorax) leaking from the thoracic duct [84]. In lungs the lymphatic marker Podoplanin [85] is not restricted to LECs, but it is also expressed by type I pneumocytes. Particularly, it seems that Podoplanin is critically involved in the proper development of the lungs. Therefore, mice lacking Podoplanin die immediately after birth due to respiratory failure, related to an impaired alveolar airspace development. They display reduced numbers of type I alveolar epithelial cells, but not type II, since their differentiation results as dysregulated and blocked. In addition, Podoplanin absence also causes a widespread impaired development of lymphatics, not correctly separating from blood vessels [86,87,88], leading to congenital lymphedema. Pulmonary lymphatic vessels also contribute to the proper lung extracellular matrix organization by maintaining hyaluronan (HA) homeostasis removing the excess of HA fragments through the endocytic receptor LYVE1 (Lymphatic Vessel Endothelial Hyaluronan Receptor 1) [89], the homologous for CD44 in blood vascular endothelial cells [90].

From a functional standpoint, the impossibility to stain the lymphatic vessels without coloring the surrounding interstitial and/or alveolar spaces is at present still a technical limit that jeopardizes every attempt to measure intraluminal pressure in situ in the intact pleural cavity. Nevertheless, measures of pulmonary *P_in_* have been performed with the intact pleural space. Results show a very negative environment, with pressures ranging from about −13.5 cmH_2_O at end-expiration to ~−20–27 cmH_2_O at end inspiration, during spontaneous breathing [91,92]. These extreme negative hydraulic pressures are a prerequisite to maintain a very thin and dry alveolar interstitial space, optimizing the gas exchange. Moreover, it is well known that the pulmonary interstitial space possesses a so-called “safety factor” [93], which is due to a very stiff extracellular matrix that forces hydraulic pressure to rapidly increase above zero when liquid tends to accumulate in the interalveolar spaces. In addition, a greater interstitial fluid volume induces anchoring filaments to stretch, thus exerting a radial tension on the lymphatic capillaries’ wall which opens primary valves increasing lymph formation [6]. The presence of a very branched and distributed network of lymphatic vessels in the lung parenchyma and in the perivascular spaces could mean that their role in the hydraulic homeostasis of the lung is not of secondary importance, as also witnessed by the aforementioned lung defects developing in KO mice or mice devoid of lung lymphatics at birth. The alveolar fluid moves to the interstitial tissue by a Na^+^-dependent transport, involving alveolar epithelium’s Na^+^ channels and Na/K-ATPase. Water binds to the extracellular matrix component HA, thus increasing *P_in_*, and then fluid clearance occurs by lymphatic vessels, according to Equation (2) [94]. Lung oedema may develop due to an increase of water permeability of blood vessels’ endothelium and/or the fluid exchange area, as well as a decrease in the blood capillary reflection coefficient to solutes, enhancing fluid filtration across blood capillaries exceeding the lymphatic transport capacity. Lung oedema increases the wet/dry ratio and values higher than ~6.5, that cannot be compensated by lymphatic reabsorption, seem to be correlated to large damages in the lung parenchyma and air-blood barrier, due to matrix fragmentation. Indeed, the glycosaminoglycans degradation to low molecular weight fragments, especially HA and chondroitin-sulphate, damages the lung interstitial scaffold dramatically decreasing the effectiveness of the “safety factor” [94,95]. Similar effects were also found to be related to the stress/strain of different ventilatory strategies, as in rat positive end-expiratory pressure (PEEP) combined with mechanical ventilation at high tidal volume (V_T_) displayed damaging effects on the lung parenchyma, due to tissue overdistension. Furthermore, injurious effects of mechanical ventilation were exacerbated by oedemagenic conditions. On the contrary, combining low V_T_ mechanical ventilation to PEEP resulted to be protective toward lung parenchyma fragmentation [95]. Moreover, PEEP would increase *P_in_*, thus favoring lymph formation and oedema reduction. Resolution of tissue oedema might be facilitated by lymphangionesis induced by exogenous VEGFC treatment, which not only increases lymphatic vessels density but also reduces oedematous-related inflammation. On the other hand, impairment of lung lymphatics and/or an excessive inflammatory-induced lymphangiogenesis and wound healing would be detrimental for lung tissue fluid balance, exacerbating interstitial tissue dehydration and facilitating the development of pulmonary fibrosis. Fibrosis consists of a pathological alteration due to an increase in fibrotic collagen deposition, dramatically affecting the lung ventilatory capability and impairing alveolar gas exchange, whose severity is critically correlated to an increase in lymphatic vessels density [96,97,98]. However, the lymphatic contribution to lung fibrosis progression it is not yet fully understood, and data are still controversial. Indeed, the opposite was found in another report, where a severe reduction in subpleural and interlobular lymphatic vessels was observed, with rare lymphangiogenesis but displaying LECs apoptosis [99].

Severe lung pathologies leading to irreversible end-stage pulmonary disease can only be addressed with a lung transplantation, which offers recipients the potential for a marked improvement in lung functionality. Due to technical limitations, transplanted lungs lack surgical reconnections between donor’s and recipient’s lymphatics: the lack of anastomosis extremely impairs lymphatic functionality. Therefore, lymph flow is hampered, and subjects often develop pulmonary oedema [100]. Despite evidence of spontaneous reestablishment of pulmonary lymph flow at a few weeks post-surgery, up to 25% of cases develop rejection and severe lung allograft dysfunctions [101,102]. However, available data seem to point out a controversial involvement of lymphatic vessels to post-surgical recovery improvement. High density of Prox1^+^ LECs is a marker of acute lung graft rejection and blocking lymphangiogenesis is well-acknowledged to dampen the recipient’s immune response to donor’s tissue [103,104]. However, therapeutic lymphangiogenesis seems to be promising for lung-graft survival, as loss of lymphatics increases low molecular weight HA aberrant deposition in rejected lung allografts. Therapeutic lymphangiogenesis ameliorates established rejection, by improving HA fragments removal and reducing inflammatory cells infiltration into allografts [105].

Lymphatic vessels can also serve as a route for primary tumor cells metastatic spread, leading to tumor metastases, which represent the primary cause of cancer-related mortality. Lymphatics in the tumor microenvironment play a heterogeneous role, since they undergo LECs proliferation and remodeling, which promote tumor dissemination to secondary lymphoid organs. The high permeable lymphatic capillaries’ wall facilitates the malignant cells evasion to regional lymph nodes, underlying the link between lymphangiogenesis and metastatic progression of many solid tumors [106,107]. In a lung cancer model, high levels of VEGFC (and VEGFD) promote cancer cells spread to regional lymph nodes and tumor lymphangiogenesis, facilitating lymphatic metastasis, including dissemination of cancer cells into the pleural space [108]. Therefore, anti-lymphangiogenic therapies seem to be promising approaches for the treatment of metastatic malignancies hampering disease progression [109,110]. Indeed, lymphatic vessels growth and lymph node metastases are reduced in a dose-dependent manner by inhibiting the VEGFR3 signaling pathway [111]. On the other hand, functional lymphatic vessels are required to develop the effective immune response, which is actively involved in the anti-tumor immunity.

The present lack of any intraluminal hydraulic pressure measurement from lung lymphatic vessels cannot allow but to speculate on their physiological properties and role in fluid drainage from lung interstitial space. From what has been observed both in the diaphragmatic and parietal lymphatics, it could be argued that during inspiration the stretch experienced by lung parenchyma could be transmitted to lymphatic vessels, being the extrinsic mechanism pivotal for pulmonary lymphatic function as lung collecting vessels lack LMCs [112]. This, in turn, might cause a more negative *P_L_*, which is the prerequisite to drain lymph from a very subatmospheric lung *P_in_*. Recently developed transgenic animal models could potentially be useful tools to close the gap in this field of lymphatic physiology [113,114,115].

Recently, few imaging techniques have been successfully applied to visualize, in vivo and in situ, lymph flow along lung lymphatic vessels. Ideally, selective labelling of the lymphatic vasculature with none or limited staining of the surrounding lung parenchyma, easy administration of contrast/staining agent and a method to achieve a flow measurement having high spatial and temporal resolution would be optimal requirements to visualize lymphatic vessels and to quantify lymph flow. Near infrared imaging with indocyanine green, coupled with a high resolution and fast acquisition device, has the potential to allow for a good spatial and temporal assessment of lymph flow [116]. A variant of this technique, employing infrared nanodots, has been successfully applied to the dynamic mapping of lymph flow towards sentinel lymph nodes of the lung [117]. However, a major drawback of near infrared imaging is that the tissue has to be in close proximity to the imaging device, so that this method can only be applied to exposed lungs to be effective. Less invasive methods make use of dynamic contrast-enhanced magnetic resonance lymphangiography, where a Gd-labelled contrast agent is injected in inguinal lymph nodes and then its progression along the lymphatic vasculature into the thoracic cavity is followed by a continuous dynamic MR acquisition of body volumes [118,119]. This technique is minimally invasive and gives a good estimate of lymph flow in lungs and thoracic regions. However, given the spatial and temporal limits of MR scanning, it cannot be applied to measure very fast lymphatic flows with high spatial resolution. Another route of administration of contrasting agents useful for imaging lymph transport in lungs is the direct inhalation of labelled solid lipid particles whose size (200 nm) and electric charge (negative) can drastically enhance the uptake by lung lymphatics [120,121]. Depending upon the labelling, magnetic resonance or CT scanning might give enough flexibility to attain a desired level of spatial and/or temporal resolution. All these methods, however, give an estimate of lymph flow but it would be very challenging if intranodal injection or tracheal instillation/direct inhalation of labelling agents could persist in the lymphatic vessels of the lung for a longer time, compatible with the micropuncture technique to directly measure *P_L_* inside labelled lung lymphatics. To this extent, near infrared imaging on exposed lungs could be the most promising.

## 6. Closing Remarks

Pleural space is a complex system from many points of view. Lubrication of the constant sliding pleurae to avoid any damage to those very thin structures, constant liquid renovation preventing excessive drying or accumulation, and the need to maintain a negative *P_liq_* for a proper lungs–chest wall mechanical coupling are overwhelming tasks, that must be fulfilled at the same time.

From the point of view of fluid drainage from interstitial space, much interest is conveyed to the pressure gradient developing between pleural liquid, subpleural spaces and the lumen of lymphatic capillaries (according to the modified Starling’s Law, Equation (2)). Direct measurement of hydraulic pressures has been performed in the past, except for those of lung lymphatics, due to still-present technical difficulties. Overall, data point to the presence of a net pressure drive to sustain liquid draining during spontaneous inspiration, where the contraction of the diaphragm and the expansion of the chest wall (and presumably the lungs) induce a significative negative shift in *P_L_*, which is still present albeit attenuated at end-expiration. Conversely, during controlled mechanical ventilation at positive airway pressure, at least in the chest wall Δ*P_TM_* is almost abolished and lymph formation is severely attenuated. This phenomenon alone might therefore partially explain mild to moderate oedematous conditions and difficulties in breathing in patients who underwent prolonged periods of mechanical ventilation.

## Data Availability

Not applicable.

## Abbreviations

CF	intrinsic Contraction Frequency
Δd	intrinsic contraction amplitude (Δdiameter)
Δ*P_Lymph_*	Intraluminal Lymphatic hydraulic Pressure gradient
Δ*P_π_*	Transmural colloidosmotic Pressure gradient
Δ*P_TM_*	Transmural hydraulic Pressure gradient
Δ*P_tv_*	Trans-Valve hydraulic Pressure gradient
HA	Hyaluronan
HC067047	selective TRPV4 channels antagonist
HCN	Hyperpolarization-activated Cyclic Nucleotide-gated channels
I_f_	Sinoatrial node “funny” current of the heart pacemaker
*J_lymph_*	Lymph Flow across the lymphatic capillary’s wall
LDLs	Low-Density Lipoproteins
LECs	Lymphatic Endothelial Cells
LMCs	Lymphatic Muscle Cells
LYVE1	Lymphatic Vessels Endothelial Hyaluronan Receptor 1
PEEP	Positive End-Expiratory Pressure
*P_in_*	Interstitial hydraulic Pressure
*P_L_*	Intraluminal Lymphatic hydraulic Pressure
*P_liq_*	Pleural hydraulic Pressure
*P_π_*_,*in*_	Interstitial colloidosmotic Pressure
*P_π_*_,*L*_	Intraluminal Lymphatic colloidosmotic Pressure
Prox1	Prospero homeobox 1
RR	Respiratory Rate
STDs	Spontaneous Transient Depolarizations
SV	intrinsic Stroke Volume
TRPV4	Transient Receptor Potential channel Vanilloid 4
VEGFC	Vascular Endothelial Growth Factor C
VEGFD	Vascular Endothelial Growth Factor D
VEGFR3	Vascular Endothelial Growth Factor Receptor 3
V_T_	Tidal Volume
